# Digital Surveillance Through an Online Decision Support Tool for COVID-19 Over One Year of the Pandemic in Italy: Observational Study

**DOI:** 10.2196/29556

**Published:** 2021-08-13

**Authors:** Alberto Eugenio Tozzi, Francesco Gesualdo, Emanuele Urbani, Alessandro Sbenaglia, Roberto Ascione, Nicola Procopio, Ileana Croci, Caterina Rizzo

**Affiliations:** 1 Multifactorial and Complex Diseases Research Area Bambino Gesù Children's Hospital IRCCS Rome Italy; 2 Paginemediche Salerno Italy; 3 Healthware Group Salerno Italy; 4 Clinical Pathways and Epidemiology Unit Bambino Gesù Children's Hospital IRCCS Rome Italy

**Keywords:** COVID-19, public health, surveillance, digital surveillance, internet, online decision support system, decision support, support, online tool, Italy, observational

## Abstract

**Background:**

Italy has experienced severe consequences (ie, hospitalizations and deaths) during the COVID-19 pandemic. Online decision support systems (DSS) and self-triage applications have been used in several settings to supplement health authority recommendations to prevent and manage COVID-19. A digital Italian health tech startup, Paginemediche, developed a noncommercial, online DSS with a chat user interface to assist individuals in Italy manage their potential exposure to COVID-19 and interpret their symptoms since early in the pandemic.

**Objective:**

This study aimed to compare the trend in online DSS sessions with that of COVID-19 cases reported by the national health surveillance system in Italy, from February 2020 to March 2021.

**Methods:**

We compared the number of sessions by users with a COVID-19–positive contact and users with COVID-19–compatible symptoms with the number of cases reported by the national surveillance system. To calculate the distance between the time series, we used the dynamic time warping algorithm. We applied Symbolic Aggregate approXimation (SAX) encoding to the time series in 1-week periods. We calculated the Hamming distance between the SAX strings. We shifted time series of online DSS sessions 1 week ahead. We measured the improvement in Hamming distance to verify the hypothesis that online DSS sessions anticipate the trends in cases reported to the official surveillance system.

**Results:**

We analyzed 75,557 sessions in the online DSS; 65,207 were sessions by symptomatic users, while 19,062 were by contacts of individuals with COVID-19. The highest number of online DSS sessions was recorded early in the pandemic. Second and third peaks were observed in October 2020 and March 2021, respectively, preceding the surge in notified COVID-19 cases by approximately 1 week. The distance between sessions by users with COVID-19 contacts and reported cases calculated by dynamic time warping was 61.23; the distance between sessions by symptomatic users was 93.72. The time series of users with a COVID-19 contact was more consistent with the trend in confirmed cases. With the 1-week shift, the Hamming distance between the time series of sessions by users with a COVID-19 contact and reported cases improved from 0.49 to 0.46. We repeated the analysis, restricting the time window to between July 2020 and December 2020. The corresponding Hamming distance was 0.16 before and improved to 0.08 after the time shift.

**Conclusions:**

Temporal trends in the number of online COVID-19 DSS sessions may precede the trend in reported COVID-19 cases through traditional surveillance. The trends in sessions by users with a contact with COVID-19 may better predict reported cases of COVID-19 than sessions by symptomatic users. Data from online DSS may represent a useful supplement to traditional surveillance and support the identification of early warning signals in the COVID-19 pandemic.

## Introduction

As of March 2021, the World Health Organization had estimated that almost 120 million cases of COVID-19 had occurred worldwide, with more than 2.5 million deaths [1]. The first cases of COVID-19 in Europe date back to January 2020, with the first 2 cases identified in Italy on January 31 in 2 Chinese tourists [2]. Since then, Italy has been severely hit by the pandemic [3]. As in other countries, from the earliest phases of the pandemic, the Italian Ministry of Health developed an information campaign with recommendations for managing contacts of SARS-CoV-2–positive individuals and information on symptoms that could suggest a possible diagnosis of COVID-19.

Despite the availability of these recommendations in the media, the surge of requests for information and advice could not be entirely managed by the health authorities through public health care helplines and family doctors. Indeed, a large part of the population tried to find appropriate answers to their questions on the web [4].

Online decision support tools have been used in the past, such as during the A/H1N1pdm09 pandemic [5], to meet the information needs of the public at large, improve measures to prevent transmission, and support the management of symptomatic cases. During the COVID-19 pandemic, online decision support systems (DSS) and artificial intelligence chatbots have also been used for digital triage and self-diagnosis and have been shown to be accurate in identifying suspected COVID-19 cases and efficient in decompressing the pressure placed on emergency rooms and helplines [6-12]. On the other hand, the adoption of digital tools encounters barriers and limitations intrinsic to their novelty, the digital divide, and other local circumstances [6,9].

To provide clinical and public health recommendations in line with the evolving guidelines issued by the Italian Ministry of Health, in February 2020, Paginemediche, a digital Italian health tech startup, developed a noncommercial, online DSS available in Italian [13]. The system was designed as a simple algorithm for assisting individuals manage their potential exposure to COVID-19 and has a chat user interface. The system was available on several web pages including landing pages of the regional health systems and was advertised through social media. A link to the online DSS was also hosted on the home page of the Regional Health Authorities of Lombardy and Campania and of Trento autonomous province.

Digital platforms collecting self-reported information have been shown to provide complementary information to traditional epidemiological surveillance [14]. In particular, it has been reported that patterns of use of online tools for COVID-19 self-triage may be considered a proxy of confirmed cases reported to national surveillance systems and may provide early signals of COVID-19 cases [15].

As our online DSS was available on a national scale from the early phase of the pandemic and for a time period of 1 year, we examined it as a potential data source for digital surveillance of COVID-19 in Italy. Our hypothesis was that trends in the use of the online DSS may precede those observed in the national surveillance system, as symptomatic individuals and those who have been in contact with a COVID-19 case may access the online DSS before undergoing a laboratory test for confirmation.

The aim of the present study was to describe the general characteristics of users of the online DSS; compare the trends in online DSS sessions with those of COVID-19 cases reported by the national health surveillance system in Italy, from February 2020 to March 2021; and study the time lag between the online DSS and the surveillance system data.

## Methods

Our online DSS was developed as a web-based algorithm to triage users with COVID-19–compatible symptoms or with a history of contact with a confirmed COVID-19 case and to provide recommendations on appropriate management (including testing), according to guidelines from the Italian Ministry of Health [16]. Anonymous access to the system is open to any user, and interaction is based on a chat interface. The system is available on the Paginemediche website [13].

Briefly, at the beginning of the session, the system collects information on age group and place of residence. Then, the user is asked to select the reason for accessing the system from among the following: (1) evaluation of symptoms, (2) contact with a confirmed COVID-19 case, (3) notification received from the national contact tracing app [17], (4) positive swab for COVID-19. The system then proceeds by matching the information provided by the users with national guidelines and provides tailored recommendations, such as asking for immediate medical support, contacting the family doctor, performing a diagnostic test, or applying nonpharmaceutical measures such as isolation and self-quarantine.

As the purpose of this study was to test if access to the online DSS preceded (and therefore was able to predict) national surveillance system trends, we restricted our analysis to users accessing the system for the evaluation of symptoms (SYM) or for contact with a confirmed COVID-19 case (CON).

We analyzed the information on users’ age and location and the symptoms reported by age group using descriptive statistics. We then explored the temporal trends in SYM and CON sessions, and we compared them with trends in COVID-19 cases officially reported by the Italian COVID-19 national surveillance system, which is based on the data from laboratory-confirmed SARS-CoV-2 infections provided daily by regional health authorities to the National Health Institute. To perform the comparison, we first used moving averages to display the curves.



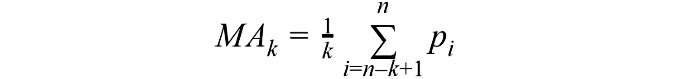



where *p_i_* are data points.

K was set at a value of 7, as data from the national surveillance system were published weekly.

We then scaled each time series in a range between 0 and 1, applying the following formula:



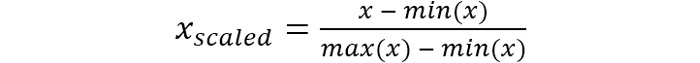



To calculate the distance between the time series of cases reported through the national surveillance system and those of SYM and CON, we used the dynamic time warping algorithm (DTW) [18,19]. DTW measures similarity between 2 temporal sequences, which may vary in speed. The sequences are “warped” nonlinearly in the time dimension to determine a measure of their similarity, independent of nonlinear variations in time.

To confirm the hypothesis that the online DSS trends anticipated those of reported COVID-19 cases, we first applied Symbolic Aggregate approXimation (SAX) encoding [20] to the time series in 1-week periods. Then, we calculated the Hamming distance [21,22] between the SAX strings. SAX transforms a time series into a sequence of symbols that represents a range of values, allowing the application of the dimensionality reduction on the time series [20,23]. SAX encoding was chosen as it allows dimensionality/numerosity reduction of the time series and as it is relatively easy to understand and compute. The dimensionality reduction was based on Piecewise Aggregate Approximation [20,22]. To verify that the online DSS anticipated the trends observed in notified cases, we shifted its time series 1 week ahead, and we measured the improvement in the Hamming distance [24]. The 1-week shift was chosen empirically based on the knowledge that the average incubation period is 4 days [25], the median time to diagnosis is 5.85 days [26], and most Italian laboratories have been providing results for PCR tests 24-48 hours after the test. Other authors have chosen the same time shift for modelling purposes [27].

This study includes data collected by the online DSS from February 25, 2020 to March 15, 2021 and the corresponding figures of COVID-19 cases in Italy published by the national COVID-19 surveillance system in their open access repository [28].

The statistical analysis was conducted using Python 3.8.5.

Considering the nature of the analysis, the current study did not require approval by the local ethics committee according to current legislation. However, a notification including the study characteristics was sent to the Bambino Gesù Ethical Committee.

## Results

We recorded a total of 75,557 sessions in the online DSS during the study period. Among them, 65,207 were SYM, and 19,062 were CON. Among the 65,207 sessions of users with symptoms, 8692 stated they also had contact with a COVID-19–positive individual.

**Figure 1:**
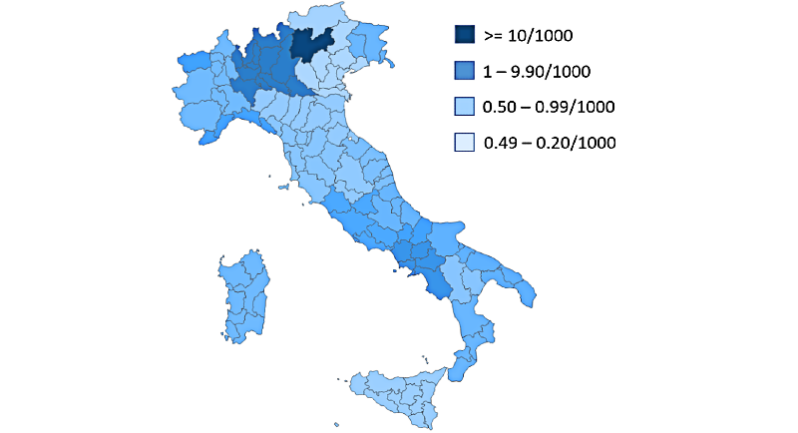
Number of sessions by the number of residents in each region.

Age was missing in nearly 32% (24,084/75,557, 31.87%) of the sessions, and place of residence was missing in 28.27% (21,360/75,557), probably because the input of information about age and place of residence was not compulsory in the first 2 months of activity in the online DSS. Among the 51,473 sessions for which age was recorded, the majority (40,923/51,473, 79.50%) were by adults in the 19–64-year-old age group: 19-24 years old, 4830/51,473, 9.38%; 25-34 years old, 9054/51,473, 17.59%; 35-44 years old, 10,383/51,473, 20.17%; 45-54 years old, 9852/51,473, 19.14%; 55-64 years old, 6804/51,473, 13.22%. Users 65 years old and older represented 12.89% (6636/51,473) of the sessions (65-74 years, 4339/51,473, 8.43%; ≥75 years, 2297/51,473, 4.46%), and 7.60% (3914/51,473) of the sessions were by users ≤18 years of age. Sessions were established from all Italian regions, at a proportion between 0.3 and 0.6 per 1000 inhabitants. The number of sessions by region are described in Figure 1. Three regions were more represented, reflecting more active local endorsement by regional authorities: 2 in Northern Italy, namely Lombardy and the Trento autonomous province, and 1 in Southern Italy, namely Campania.

Symptoms reported by online DSS users by age group are illustrated in Table 1.

Symptoms reported by users varied with age. In all age groups, fever was the most reported symptom, although the proportion of patients reporting fever was higher in younger individuals and decreased with age. A similar trend was observed for respiratory symptoms, such as cold and sore throat. Among other symptoms, myalgia, low blood pressure, and dizziness were most frequently reported among people belonging to older age groups.

**Table 1:** Distribution of symptoms reported in each session by age group (n=51,473).

Symptom	Age (years), n (%)			
	0-18 (n=3914)	19-64 (n=40,923)	65-74 (n=4339)	≥75 (n=2297)
Fever	1912 (48.85)	12,896 (31.51)	993 (22.89)	625 (27.21)
Cold	1489 (38.04)	9584 (23.42)	801 (18.46)	406 (17.68)
Sore throat	1027 (26.24)	10,264 (25.08)	628 (14.47)	305 (13.28)
Cough	881 (22.51)	8540 (20.87)	831 (19.15)	441 (19.20)
Headache	437 (11.17)	4870 (11.90)	280 (6.45)	82 (3.57)
Myalgia	262 (6.69)	4947 (12.09)	643 (14.82)	284 (12.36)
Dispnea	381 (9.73)	4296 (10.50)	362 (8.34)	314 (13.67)
Low blood pressure	93 (2.38)	2612 (6.38)	611 (14.08)	322 (14.02)
Dizziness	301 (7.69)	2504 (6.12)	298 (6.87)	251 (10.93)
Drowsiness	214 (5.47)	2195 (5.36)	269 (6.20)	220 (9.58)
Tachycardia	260 (6.64)	2043 (4.99)	265 (6.11)	138 (6.01)
Nausea	285 (7.28)	1947 (4.76)	157 (3.62)	61 (2.66)

**Figure 2:**
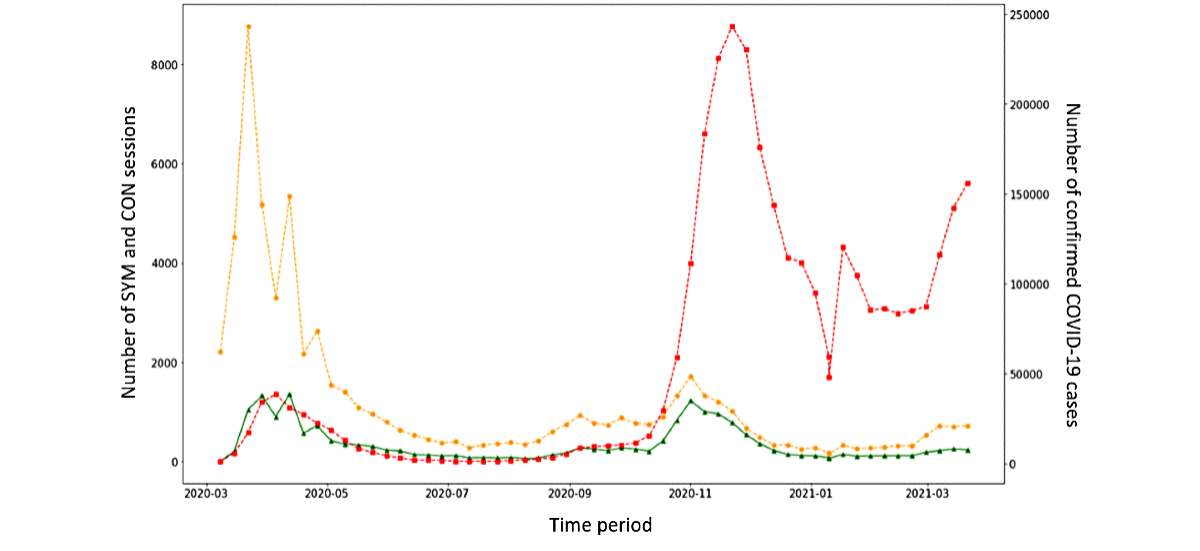
Weekly number of COVID-19 confirmed cases (red), SYM (symptomatic users) sessions (yellow), and CON (users with contact with a COVID-19 case; green) sessions between February 2020 and March 2021.

Figure 2 shows the numbers of SYM and CON sessions over time and the number of confirmed COVID-19 cases officially reported to the national surveillance system.

The highest number of sessions was recorded in the early phases of the pandemic, when COVID-19–confirmed cases started to increase very quickly.

**Figure 3:**
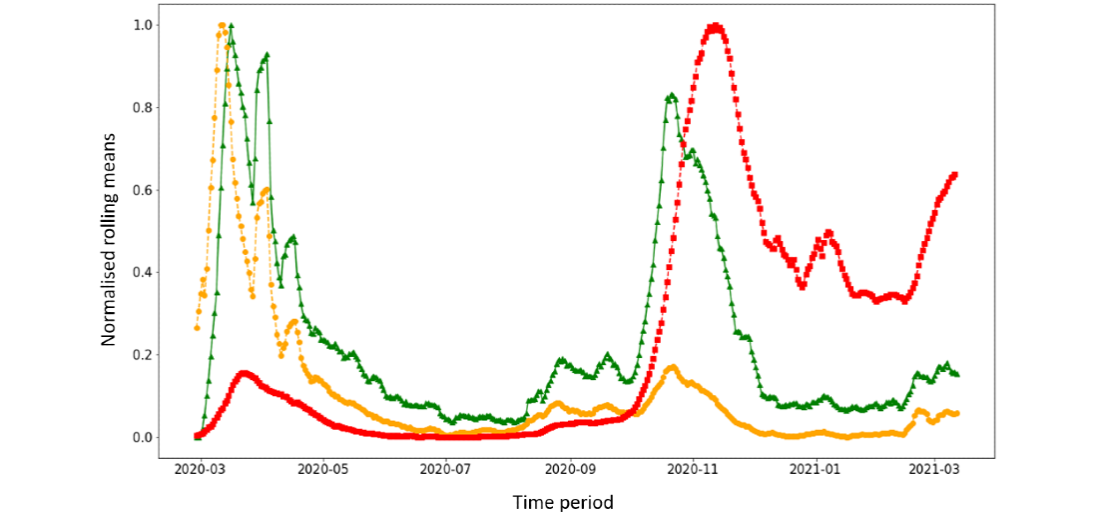
Time trends in SYM (symptomatic users) sessions (yellow), CON (users with contact with a COVID-19 case) sessions (green), and COVID-19 cases (red). Values are illustrated as rolling means in 1-week periods and scaled to values between 0 and 1.

A less pronounced peak was observed in the number of CON sessions in the same period. A second peak, both in SYM and CON sessions, was observed during a large second wave of COVID-19 cases, which, in Italy, started in October 2020. Finally, a third peak was observed in March 2021, when Italy experienced a third surge of cases. Figure 3 shows the trends in SYM sessions, CON sessions, and COVID-19 cases as moving averages in 1-week periods and scaling values between 0 and 1.

The 3 time series in the entire time period had a similar trend, independently from the amplitude of the peaks, although an increase in SYM and CON anticipated the trend in confirmed COVID-19 cases by approximately 1 week. The distance between SYM and notified COVID-19 cases calculated through DTW (the lower the distance, the higher the similarity between the curves) was 93.72, while that between CON and notified COVID-19 cases was 61.23.

**Figure 4:**
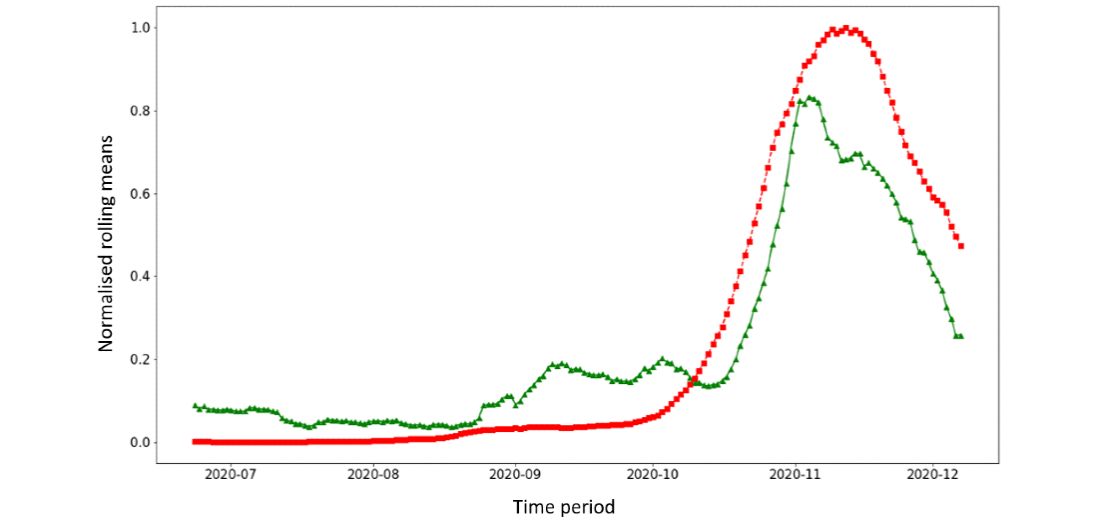
Time trends in CON (users with contact with a COVID-19 case) sessions and notified COVID-19 cases, Italy, July 2020 to December 2020. Values are illustrated as rolling means and scaled from 0 to 1. The CON time series is shifted 7 days ahead.

As CON was more consistent with the trend in confirmed cases than SYM, we tested the hypothesis that this series anticipated confirmed cases by 1 week. After applying SAX encoding, we measured the Hamming distance between the time series of confirmed COVID-19 cases and the CON series (1) as recorded by the system and (2) shifted by 1 week. After applying the 1-week shift, the Hamming distance improved from 0.49 to 0.46. We also repeated the analysis restricting the time window to the time period between July 2020 and December 2020. The corresponding Hamming distance was 0.16 before shifting the time series and improved to 0.08 after the time shift (Figure 4).

## Discussion

In this study, we showed that users of a national online DSS reporting contact with a COVID-19 case anticipated trends observed by the Italian national surveillance system by 1 week.

Surveillance systems for infectious diseases frequently suffer from lack of timeliness and difficulties in case finding that may delay the implementation of effective, data-based preventative strategies [29,30]. Digital surveillance has been indicated as a potential supplement to traditional surveillance due to the fact that web and social media users may spontaneously leave online traces related to their health conditions, which can be detected by digital surveillance systems in a timely manner [31].

Classically, digital traces for surveillance are classified as either based on the public’s demand of information (eg, volume of searches on Google) or on information supplied by internet and social media users (eg, tweets reporting symptoms of a specific disease) [32]. Indeed, several attempts have been made to track and interpret the signals from queries on search engines or from keywords or symptoms reported on social media to promptly recognize the emergence of infectious diseases, with mixed success [33]. As a matter of fact, trends in queries on search engines are strongly affected by media coverage that may not accurately reflect the epidemic trends [34].

With this study, we explored the potential of a digital surveillance system based on a mixed demand-supply method. Users accessed our online DSS with a specific request for information and were invited to provide specific data on their condition through a structured framework. The algorithm guided the user through predefined questions based on national guidelines for COVID-19 prevention and contact management, with the aim to provide tailored, actionable recommendations.

Our results show that temporal trends in the number of sessions on an online COVID-19 DSS dedicated to the general public may precede the trend in confirmed COVID-19 cases obtained through the national surveillance system by 7 days. We found a high correlation between the trend in sessions by users accessing the online DSS (with either a COVID-19 contact or symptoms) and the number of notified COVID-19 cases, documented by the short distance between the trends as estimated with DTW. However, the trends in sessions by users who had contact with COVID-19 cases predicted the trend in COVID-19 cases better than sessions by users with symptoms. This observation is plausible as, on the one hand, recognition of contact with someone with COVID-19 usually precedes the onset of symptoms, and, on the other hand, contact with a COVID-19 case can be considered as a more specific proxy of COVID-19, as the clinical picture of COVID-19 may overlap with that caused by other diseases. The correlation between sessions by users with a COVID-19 contact and confirmed cases greatly improved when the analysis was restricted to the central phase of the pandemic, from July 2020 to December 2020. In this time period, Italy experienced the highest surge of COVID-19 cases.

To our knowledge, only one other study attempted to use sessions in an online DSS as a proxy for case notification [15], showing lead times of 3 days in China and 19 days in the United States. Although the recognition of contact with a COVID-19 case may be delayed, our estimate of a 7-day lead time from users with contact with a COVID-19 case is consistent with the incubation of COVID-19 [26] and the time needed to perform a diagnostic test and obtain results.

These results show that the utility of an online DSS dedicated to the general public may go beyond its original purpose of guiding the public through recommendations for the management of cases or contacts.

First, these systems may generate early warning signals that may inform early interventions (eg, tailored restriction measures), as is desirable during the current pandemic. Data from our system may be particularly valuable in predicting large and rapid increases in COVID-19 cases, as shown by the higher correlation between the trend in contacts and national notifications.

Second, the high correlation estimates obtained through DTW support the inclusion of online DSS–based data in the development of prediction models, which could be useful to anticipate an increase in cases as well as to predict the timing of a reduction in cases. This would support national or regional institutions in more finely programming (timing, intensity, logistics, economic impact) the restrictive measures to contain the epidemic.

Digital data have already been used in prediction models for influenza. Through the analysis of the volume of online searches for flu-related terms, Google Flutrends offered a forecast of the trend in influenza-like illness cases in the United States [35], and a similar approach has recently been used in the Netherlands [36]. Data for participatory surveillance have also been integrated with classic surveillance data [37]. Although prediction models including participatory surveillance data can suffer from a lack of representativeness of the population sample, modeling and simulation can help control this bias [38].

In a recent study, Kogan et al [39] attempted to combine and harmonize different digital data streams to predict COVID-19 trends. They included Google Trends, Twitter, UpToDate, and mobility data, but no data from online DSSs were taken into account, which could have potentially improved the performance of the model.

For the purpose of informing prediction models with digital surveillance data, online DSS data would benefit from integration with data from contact tracing apps. Contact tracing apps have been developed in several countries, including Italy, to support public health measures for COVID-19 containment. These digital tools send a notification of exposure to a COVID-19–positive case to contacts and recommend preventative actions. However, data about the number of contacts and other personal characteristics remain stored in personal smartphones and cannot be used as a data source for surveillance. Moreover, although Italy has been considered as one of the countries with the highest acceptance of apps for contact tracing [40], only 20% of the general population actually downloaded the app as of March 2021 [41].

In this article, we report national data obtained through our online DSS. However, it should be noted that a very high user rate has been recorded in the Lombardia and Campania regions and in the autonomous province of Trento. The potential of predicting actual COVID-19 trends was more accurate in areas with a higher coverage of the system. In these regions, the online DSS had been actively promoted by local health institutions. The involvement of health authorities and the promotion of these systems by digital marketing through online social media are likely to increase the use of these digital tools, underlying their value in providing tailored recommendations to individuals. Stronger involvement of health authorities, by increasing participation and accuracy, would also allow us to develop prediction models on a regional — rather than on a national — basis.

This study has several strengths. First, data were available over a 12-month period on a national scale. We also found that the online DSS was accessed from all Italian regions, which was reassuring for the representativeness of the data sample. The system was also open to any user, as there was no age restriction for accessing the system.

The study also has obvious limitations. The most important is that we could not validate information provided by users, as expected from any anonymous online tool. Moreover, the number of sessions in an online DSS like ours may be affected by different factors that may increase the background noise and make the interpretation of trends difficult. Some of the fluctuations observed in the use of the online DSS may reflect a different perception of risk during different phases of the pandemic. Indeed, infodemics may affect perception of risk of infection that may result in an increase in online searches for information by the general public [42]. We cannot exclude that users’ access to our online DSS might have been biased by different trends in interest by the general public, possibly affected by different media coverage of the pandemic throughout the study period. Nevertheless, the high correlation between our access data and the epidemic curve suggests that the variation of users’ interest and, subsequently, of their online searches has not had a major impact on the use of our online DSS, which seems to have been primarily affected by the disease incidence. Nevertheless, in order to better validate a system like the one proposed in our study, future research should better estimate the impact of online searches on access to the online DSS, possibly correcting the analysis for the volume of searches based on keywords related to the epidemic.

Moreover, especially in the early phase of the pandemic, users may have tried to simulate different scenarios to better understand what to expect in case of symptoms or contact with a COVID-19 case. As a matter of fact, when restricting the analysis by excluding the earliest phase of the pandemic, the consistency of the online DSS time series with confirmed COVID-19 cases was higher. Furthermore, we did not collect socioeconomic information, and we could not attempt to adjust our analyses for confounders. Finally, all data streams from digital systems may suffer from selection bias due to the digital divide [43]. As a matter of fact, only 13% of the sessions were by individuals over the age of 65 years, which was the age group mostly affected in the first phase of the pandemic. Therefore, we should consider that this age group may have not been well represented in the online DSS sessions, and this might have affected the accuracy of our prediction. Nevertheless, we were not interested in estimating the incidence by age group based on the online DSS data, as our main objective was to study the trends in access to the online DSS and their relationship with official surveillance data. The high number of sessions recorded by any age group and from any region and their consistency over time with the trend in notified cases are reassuring that potential major biases are not present.

In conclusion, as online DSSs like the one described in this study are widely available and may be completely anonymous, they may represent a significant source of data to inform public health actions, may supplement other epidemiological information collected through other means, and may help to develop more accurate prediction models for pandemic trends.

Public health strategies for immunization and containment are usually based on incidence of preventable diseases and vaccine coverage, which carry some latency due to the notification systems. Despite the observed limitations, prediction models based on systems like the one described in our study may anticipate other epidemiological signals of epidemic surge and, when integrated with official surveillance data, can support timely decisions for mitigating the pandemic spread.

Digital tools for managing COVID-19 not only have epidemiological implications but also may be extremely helpful in disseminating appropriate recommendations to the general population in a universal strategy.

Although it remains to be assessed how frequently recommendations provided are actually followed by users and selection bias should be better understood and addressed, these tools represent a potential data source for public health that may help, in combination with others, to expeditiously implement containment strategies.

## Abbreviations

CON: users accessing the system because of contact with a confirmed COVID-19 case
DSS: decision support system
DTW: dynamic time warping
SAX: Symbolic Aggregate approXimation
SYM: users accessing the system for the evaluation of symptoms
